# Attributable Burden and Expenditure of Cardiovascular Diseases and Associated Risk Factors in Mexico and other Selected Mega-Countries

**DOI:** 10.3390/ijerph16204041

**Published:** 2019-10-22

**Authors:** Kenny Mendoza-Herrera, Andrea Pedroza-Tobías, César Hernández-Alcaraz, Leticia Ávila-Burgos, Carlos A. Aguilar-Salinas, Simón Barquera

**Affiliations:** 1 Center for Nutrition and Health Research, National Institute of Public Health, Cuernavaca, Morelos 62100, Mexico; cinys26@insp.mx (K.M.-H.); cesar.hernandez@insp.mx (C.H.-A.); 2 Institute for Global Health Sciences, University of California, San Francisco, CA 94158, USA; Andrea.Pedroza-Tobias@ucsf.edu; 3 Center for Health Systems Research, National Institute of Public Health, Cuernavaca, Morelos 62100, Mexico; leticia.avila@insp.mx; 4 Unidad de Investigación de Enfermedades Metabolicas, Mexico City 14080, Mexico; caguilarsalinas@yahoo.com; 5 Departamento de Endocrinología y Metabolismo, Instituto Nacional de Ciencias Médicas y Nutrición Salvador Zubiran, Mexico City 14080, Mexico; 6 Tecnologico de Monterrey, Escuela de Medicina y Ciencias de la Salud, Monterrey 64710, N.L., Mexico

**Keywords:** Mexico, mega-countries, cardiovascular diseases, burden, expenditure

## Abstract

Background: This paper describes the health and economic burden of cardiovascular diseases (CVD) in Mexico and other mega-countries through a review of literature and datasets. Methods: Mega-countries with a low (Nigeria), middle (India), high (China/Brazil/Mexico), and very high (the U.S.A./Japan) human development index were included. The review was focused on prevalence of dyslipidemias and CVD economic impact and conducted according to the PRISMA statement. Public datasets of CVD indicators were explored. Results: Heterogeneity in economic data and limited information on dyslipidemias were found. Hypertriglyceridemia and hypercholesterolemia were higher in Mexico compared with other countries. Higher contribution of dietary risk factors for cardiovascular mortality and greater probability of dying prematurely from CVD were observed in developing countries. From 1990–2016, a greater decrease in cardiovascular mortality in developed countries was registered. In 2015, a CVD expense equivalent to 4% of total health expenditure was reported in Mexico. CVD ranked first in health expenditures in almost all these nations and the economic burden will remain significant for decades to come. Conclusions: Resources should be assured to optimize CVD risk monitoring. Educational and medical models must be improved to enhance CVD diagnosis and the prescription and adherence to treatments. Long-term benefits could be attained by modifying the food system.

## 1. Introduction

Currently, cardiovascular diseases (CVD) are considered a major global health problem [1]. The global cumulative economic loss from not implementing strategies to tackle CVD and other chronic diseases from 2010 to 2030 is expected to be $47 trillion US dollars (USD), which is equivalent to 75% of the 2010 gross domestic product (GDP) [2,3,4,5]. Due to the health and economic burden that these diseases generate, the World Health Organization (WHO) launched an action plan for prevention and control of chronic diseases for 2013–2020, creating a global initiative with a series of strategies to reduce the negative effects of CVD [3].

The high prevalence of obesity and non-communicable diseases (NCDs), as well as the increased mortality from these causes since the nineties, have placed Mexico in the spotlight of global health [6,7,8]. National health surveys in Mexico have identified dyslipidemias as the second most prevalent modifiable cardiovascular risk factor in adults, just after a body mass index (BMI) above 25 kg/m^2^ [9]. As in other countries, a high low-density lipoprotein cholesterol (LDL-c) level has been described as the second most common risk factor for cardiovascular mortality in Mexico [10]. Since dyslipidemias are major contributors to the development of atherosclerosis [11], one of the main pathophysiological paths of CVD, their prevention and treatment are essential to counteract CVD burden and related expenditures. In order to direct strategies and allocate human and monetary resources to diminish the burden of lipid alterations, surveillance systems and studies that show the epidemiological status related to this health issue are needed.

Environmental factors have a crucial influence on the economic and health burden of CVD. As in other countries [12,13], the Mexican food system has undergone several transformations in recent decades. These modifications have caused a deterioration in diet, characterized by an increase in the consumption of ultra-processed food, sugar-sweetened beverages, and a low intake of fruits, vegetables, and whole grains [14,15,16,17,18,19]. This scenario has been associated with the increased prevalence of obesity, dyslipidemias, diabetes, and CVD, which represent important challenges for the health system and economy [3,8,9,12,13,14,20].

In Mexico, CVD represent the leading cause of mortality, accounting for ≈20% of total deaths, from which 68.5% are caused by ischemic heart disease (IHD). Estimates indicate that 4% of total health expenditure in 2006 was spent on addressing CVD. Furthermore, investment in prevention strategies is much lower than in treatment and control actions [2]. Seventy-three percent of diabetes, obesity, and CVD expenditures are absorbed by three Mexican public health institutions: the Ministry of Health, the Mexican Social Security Institute (Spanish acronym: IMSS), and the Institute for Social Security and Services for State Workers (Spanish acronym: ISSSTE).

Sixty-five percent of global CVD mortality is concentrated in Mexico and the other 13 most populated mega-countries in the world. Among these countries, a great cultural diversity, distinct economic potential, and different epidemiological and nutrition transition stages can be observed. For example, although risk factors for NCDs, such as overweight and obesity, are not as much prevalent in Nigeria and India as in other mega-countries, even a low national prevalence of these conditions represents an enormous absolute number of affected people since they are two of the most populous countries in the world. Brazil, China, and Mexico, which are mainly dominated by a high prevalence of obesity and other risk factors, currently have economic systems that discourage healthy food systems and accelerate urbanization, thereby producing substantial changes in lifestyles in the last decades. The U.S.A. and Japan, two of the most developed mega-countries, are also experiencing a significant prevalence of NCDs. However, these two countries have been successful in decreasing the burden of some cardiovascular risk factors, and they have significantly more economic resources to address public health issues than other nations from this group [12].

Despite these differences, these countries have common political and economic characteristics that influence how the food and health system are regulated, which inevitably feed the global NCDs epidemic [12,21,22]. Because of this, understanding the dynamics of CVD-related epidemiological and economic scenarios in Mexico can contribute to identify effective strategies and critical financial measures to diminish the burden associated with these conditions. In addition, exploring and comparing this situation among other mega-countries can also be useful for visualizing reproducible solutions in this group of nations. Thus, this paper aims to describe the burden and national economic impact of CVD in Mexico, as well as to complementary compare these indicators from other selected mega-countries through a review of published literature and the exploration of secondary datasets.

## 2. Materials and Methods

A literature review and exploration of secondary datasets with epidemiological indicators of CVD and their risk factors were carried out, in addition to a review on the expenditure generated by its medical costs in Mexico. Data from six selected mega-countries with a population of ≥100 million [23] and a low (Nigeria), middle (India), high (China, Brazil, and Mexico), or very high (the U.S.A. and Japan) human development index (HDI) were also included. The HDI is composed of three indicators: (1) Health that considers life expectancy at birth; (2) education that considers the adult literacy rate and years of education; and (3) income with GDP per capita in international dollars. Thus, HDI allows for a comprehensive comparison between countries [24].

### 2.1. Literature Review

A literature review was conducted and reported following the standards provided by the PRISMA statement [25,26] (PRISMA checklist (see Supplementary file 1)). Searches were first carried out in PubMed. Afterwards, to complement the review, a second search was performed considering the following order: ScienceDirect, Google Scholar, ResearchGate, and reference lists from studies. Duplicated studies found in the different stages were excluded from the final count. The review was conducted on two main topics: (1) Prevalence of dyslipidemias and (2) attributable economic burden to CVD, both nationwide. This review was carried out between January 2017 and December 2018.

The search period was limited to 2006 due to the availability of information on CVD costs in Mexico. As a result, to provide a standardized description of both topics, the first general inclusion criterion for the identification process was literature published in and related to the period between 2006 and 2018. The same search period was applied for the rest of the countries.

Identification, screening, filtering, and eligibility processes were performed for each topic. Searches were also limited to the availability of full abstracts and studies in humans. Search strategies were composed mainly of MeSH terms, but free vocabulary was also considered. Language restriction filters were not applied in the identification and screening processes. Eligibility and inclusion of studies were restricted to either English or Spanish, which are languages with which authors are familiar. Studies without clearly reported methods were excluded. Search strings are available in Supplementary Table S1 (see Supplementary file 2).

Given the implications of dyslipidemias for cardiovascular risk and the importance of actions against these lipid alterations to reduce CVD burden and expenditures described above, the first search was focused on the prevalence of high triglycerides (TG), high total cholesterol (TC), low high-density lipoprotein cholesterol (HDL-c), and high LDL-c in healthy adults ≥18 years old. The terms used in this review were “dyslipidemia” (or “dyslipidemias”) and “prevalence” (or “epidemiology”). Additionally, titles and abstracts of the identified articles were screened and filtered looking for the terms of lipid alterations, lipid abnormalities, proportion, patterns, trends, and population-based survey. For the eligibility process, the order of the following inclusion criteria and study characteristics were considered: (1) The most recent population-based surveys nationwide; (2) the most recent metanalyses or systematic reviews nationwide; (3) the most recent population-based surveys at regional level, and (4) studies with samples from multiple cities. Studies and information related to comorbidity of dyslipidemias with other conditions were excluded.

The second search was concentrated on estimates associated with economic burden attributable to CVD. CVD included in the 10th revision of the International Statistical Classification of Diseases and Related Health Problems (ICD-10) and their risk factors were considered in this stage. The terms used on this search were “cardiovascular disease” (or “cardiovascular diseases”) and “cost” (or “economics”). The following vocabulary was considered for screening and filtering of abstracts and titles: expenditure, expenses, charges, spending, spent, economy, economic burden, economic impact, coronary heart disease (CHD), angina, heart disease, ischemic heart disease, myocardial infarction, myocardial ischemia, stroke, cerebral ischemic vascular event, ischemic vascular event, and vascular event. The main outcomes for this review were expenditures, rank of economic impact among different causes, fraction of relevant economic indicators spent on CVD, and projected economic losses and expenses. Full texts of cost-effectiveness studies were not reviewed. The following inclusion criteria were considered in the eligibility process: (1) Nationwide information; (2) report of monetary indicators, and (3) use of cost components, cost of illness methods, and modelling approaches. Microeconomic data were excluded (e.g., cost of treatment per patient year).

Identification and screening processes were carried out by one trained researcher. Afterwards, each potential article was fully reviewed in order to fill out the inclusion criteria checklist and to exclude studies that did not meet the criteria. All the studies classified as eligible were read by the research team and their inclusion decided by consensus. Data on authors, titles, databases, study design, year of estimation, main indicators, reported methodology, and main outcomes of the reviewed and included studies were extracted and summarized.

In order to standardize economic information, estimate expenditures from Mexico were deflated by considering the accumulated inflation until the year of the most recent information: 2006−2016 = 46.81%; 2008−2016 = 32.82%; 2014−2016 = 5.56%, and 2015−2016 = 3.36%. Additionally, these estimates were adjusted by purchasing power parities (PPP) for 2016 (1 USD-PPP = $8.87 pesos). Meanwhile, Mexican economic projections are presented as they appear in the literature. Economic data in currencies from other mega-countries were calculated to USD using the average yearly exchange rate. In addition, data from the studies that provide estimates in USD were kept and presented in this way.

### 2.2. Review and Analysis of Secondary Databases

Databases from the Mexican national surveys of health and nutrition (Spanish acronym: ENSANUT) 2006 and 2012 [27,28] were analyzed to estimate the prevalence of obesity and hypertension in the northern, central-western, central, and southern regions of Mexico. Datasets of medicines purchases from the IMSS were reviewed in order to obtain information of expenditure on statins in Mexico [29].

Data from the Global Burden of Disease (GBD) study 2017 [10] related to age-standardized mortality rates of CVD and the attributable percentage of metabolic and dietary risk factors to cardiovascular deaths were reviewed. The metabolic risk factors were high systolic blood pressure, high LDL-c, high fasting plasma glucose, excess body weight, and impaired kidney function. The dietary risk factors were a low intake of nuts/seeds, vegetables, fruits, legumes, whole grains, fiber, polyunsaturated fatty acids (PUFAs) Ω-3 from fish and seafood, a high intake of trans fatty acids, sodium, processed meat, and sugar-sweetened beverages. In the case of Mexico, information on IHD, ischemic stroke, CVD in general, and all the previous risk factors were considered. For the other selected countries, data on only CVD in general and four risk factors were included: (1) Low intake of seeds and grains; (2) low intake of polyunsaturated fatty acids (PUFAs) Ω-3; (2) hypertension; and (3) hypercholesterolemia.

The WHO Global Health Observatory databases [30] were explored. From these, the indicator that reflects the probability of dying between 30 and 70 years old (premature death) from any CVD, cancer, diabetes, or chronic respiratory disease, was obtained. The information was reviewed for 2000 and 2016 in the seven mega-countries.

A narrative synthesis of the information identified in the literature reviews and the secondary datasets was conducted. In order to complement this article, some strategies to decrease the burden of CVD were discussed (no extensive review was performed for this particular literature).

## 3. Results

### 3.1. Literature Search

**Figure 1:**
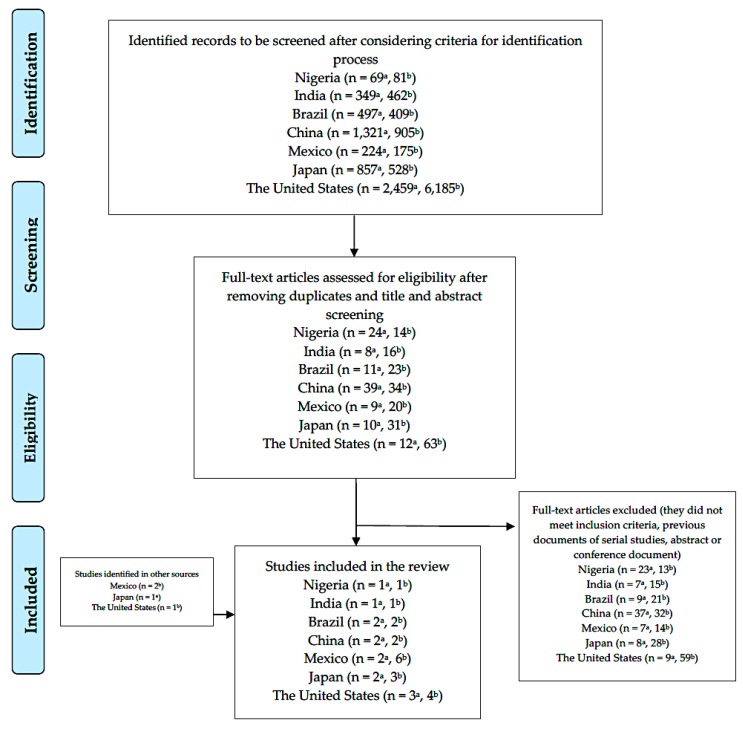
PRISMA flow diagram, literature review for seven mega-countries. a Search for dyslipidemias, b search for economic burden of cardiovascular diseases.

The detailed PRISMA flow diagram for the reviews on prevalence of dyslipidemias and CVD economic burden in seven mega-countries can be visualized in Figure 1. After removing duplicates, a total of 5776 studies were found on prevalence of dyslipidemias in the analyzed mega-countries. One hundred thirteen articles were fully reviewed and 13 of them were included. The number of identified studies on the economic burden of CVD in these nations was 8745 after duplicates, from which 201 were fully read and 19 included in this review. A summary of all the fully reviewed and included studies can be found in the Supplementary Tables S2–S5 (see Supplementary file 2).

The nationwide and regional representative prevalence of hypertriglyceridemia, hypercholesterolemia, hypoalphalipoproteinemia, and high LDL-c, as well as the prevalence of Mexican adults eligible for pharmacological and lifestyle lipid-lowering therapy according to cardiovascular risk profiles in 2006, were obtained from two studies derived from ENSANUT [9,31]. Eligibility profiles in the latter study were based on lipid-lowering treatment recommendations considering the absolute risk of having CHD according to the National Cholesterol Education Program (NCEP) and the sex- and age-specific Framingham score tables [31,32].

The most recent information about the prevalence of dyslipidemias in the U.S.A. [33,34,35] and Japan [36,37] was obtained from studies based on analyses of the National Health and Nutrition Examination Survey (NHANES) and from a review on the Japanese National Health and Nutrition Survey 2015. For India, evidence of 2008–2010 from the Indian Council of Medical Research India Diabetes Study (ICMR-INDIAB study) was included [38]. Data from Nigeria were obtained from a study based on population-based surveys of four geopolitical areas [39], and data from China came from a 2014 metanalysis [40] and a nationally representative survey carried out between 2013 and 2014 [41]. In the case of Brazil, this information was collected from a prospective study with 15,105 participants [42] and the prevalence of self-reported high cholesterol levels was identified in a study based on the National Health Survey 2013 [43].

Expenditure estimates of CVD in Mexico were identified in a report from 2006, which included information on curative medical care, medical products, prevention actions, health management, laboratory services, research, and technological development in the Ministry of Health, IMSS, ISSSTE, and the private sector [2]. Estimates from the Ministry of Health and IMSS related to expenses on medical consultation, laboratory tests, medications, and hospitalization from addressing hypertension and IHD nationwide were obtained from a 2012 ENSANUT study [44].

Information on the expenditure generated by five CVD in Mexico in 2015 was identified through a study in press. These estimates considered the economic burden of medical treatment, and formal and informal medical care in the Mexican health system. In addition, they included the loss of productivity and welfare caused by hypertension, acute myocardial infarction, atrial fibrillation, and heart failure nationwide [45].

Expected total costs associated with hypertension for 2016 in Mexico, including indirect and direct expenditures for the Ministry of Health, IMSS, ISSSTE, and patients and families were identified in a study published in 2015 [46]. These estimates were based on national registries and were calculated through a modelling approach considering the changes in cases and demand of services in the health system. Another study from 2017 with similar sources and methodology was included. In this article, estimates concerned only older adults in Mexico [47]. Additionally, information on IMSS expenditures on atorvastatin and pravastatin in 2016 was obtained from the institution’s online datasets of medicine purchases. These statins are part of the official catalogue of drugs from the Mexican public health sector.

Projections of the attributable expenditure to IHD, acute myocardial infarction, and stroke in Mexico were identified in a 2014 study. These estimates were based on USD from 2010 and three hypothetic scenarios of obesity rate changes between 2010 and 2050: (1) No reduction of the average BMI in the Mexican population; (2) reduction of 1.0%, and (3) of 5.0% [48,49,50,51]. These trends were based on BMI data from Mexican health and nutrition surveys of 1993, 1999, 2000, and 2006.

Data on the national economic burden of CVD in the other mega-countries were collected from a series of studies and reports [52,53,54,55,56,57,58,59,60,61,62,63,64,65,66,67,68,69,70,71]. Beginning with countries of a low and middle HDI, data of projected economic losses due to CVD in India between 2012 and 2030 were identified in a study based on the World Health Organization’s EPIC model of economic growth. Information from Nigeria was obtained from a study that aimed to estimate the expected loss of GDP to 2015 in 23 low- and middle-income countries through an economic growth model.

Two studies related to CVD expenditures in China were included. The first one was related to estimates of foregone GDP projected to 2030 based on a human capital augmented production function, and the second one to hospitalization expenses in 2014. The included studies for Brazil concerned the economic impact of hypertension, heart failure, myocardial infarction, and atrial fibrillation including direct and indirect costs calculated through a cost of illness framework, as well as estimates on direct and indirect cost of CVD nationwide in 2015.

Evidence of CVD economic impact for mega-countries with a very high HDI were identified in seven sources. Expenditure estimates on ambulatory, emergency, inpatient, and nursing facility care, as well as pharmaceuticals in 2013 for the U.S.A were identified in one study. Data of direct and indirect costs between 2013 and 2035 were found in two reports from the American Heart Association. Current and projected estimates associated with informal caregiving for patients with CVD were obtained from another article (2015–2030, zero-inflated binomial model). For Japan, information on expenditures generated by direct medical care, morbidity and mortality, as well as by expenditures from insurance benefits and those incurred by family and friends for medical care at the household or community level, was identified in a single study. Other study based on the Cost of Illness method and modelling estimations reporting the economic impact of IHD in 2014, and between 2017 and 2029, was also included. In addition, projections of foregone GDP to 2030 based on a human capital augmented production function were identified.

A total of 16 reports and studies about strategies against CVD were reviewed for discussion. The included interventions were those identified by the WHO as the most cost-effective [3] and other similar strategies studied in isolation among the selected countries.

### 3.2. CVD Risk Factors

After obesity, hypoalphalipoproteinemia (HDL-c < 50 mg/dL for women and <40 mg/dL for men) and high LDL-c (≥100 mg/dL) were the most prevalent cardiovascular risk factors in Mexican adults in 2006 (Table 1). In the same year, the prevalence of hypercholesterolemia in northern and central Mexico was higher than in the other regions.

Prevalence of hypertension (systolic/diastolic blood pressure ≥140/≥90 mm Hg) in Mexico did not show substantial changes in the analyzed period. This condition was observed in three out of every ten Mexican adults and its prevalence in the northern region was the highest in 2006 and 2012. According to the newest data from the ENSANUT, prevalence of high blood pressure in Mexican adults was 25.5% in 2016 (data is not shown on tables) [72].

The most recent prevalence of hypertriglyceridemia and hypercholesterolemia in Mexico was higher compared with that in India, Nigeria (TG ≥ 150 mg/dL and TC ≥ 200 mg/dL), China, Japan (TG ≥ 200 mg/dL and TC ≥ 240 mg/dL), and in the U.S.A. (TG ≥ 150 mg/dL and TC ≥ 240 mg/dL) (Table 1 and Table 2). The highest prevalence of high LDL-c were found in Brazil (57.6% (women) 58.5% (men), LDL-c ≥ 130 mg/dL), Mexico (46.0%, LDL-c ≥ 130 mg/dL), and in the U.S.A. (27%, LDL-c ≥ 160 mg/dL (low risk groups), ≥130 mg/dL [intermediate-risk groups], and ≥100 mg/dL (high risk groups)). India had the highest prevalence of hypoalphalipoproteinemia (<50 mg/dL for women and <40 mg/dL for men).

### 3.3. Prevalence of Eligible Mexican Adults for Lipid-Lowering Therapy

In 2006, 36.3% of Mexican adults were eligible for therapy of lifestyle changes and 24.2% for pharmacological treatment considering their cardiovascular risk profile (Table 3) [31]. From the Mexican adults with cardiovascular risk profile one, 70.5% were eligible to follow pharmacological and lifestyle treatment. From the adults with diabetes, 71.4% were candidates for both treatments. Around 39.0% of the adults with two or more cardiovascular risk factors were candidates for lifestyles changes and 23.9% for medicines. The prevalence of Mexican adults with cardiovascular risk profiles two, three, and four that were candidates for treatment of lifestyle changes was 30.7%, 55.3%, and 80.5% respectively. Meanwhile, the prevalence of adults with these profiles that required drugs was of 10.9%, 55.3%, and 80.5%, respectively [31].

### 3.4. CVD Mortality Attributable to Metabolic and Dietary Risk Factors

According to the GBD study in 2017, dietary risks factors contributed 48.4% to total CVD deaths, and 65.7% and 23.2% to the mortality associated with IHD and ischemic stroke in Mexico, respectively (Table 4). The intake of trans fatty acids contributed 6.7% to total CVD deaths and 10.2% to IHD deaths in the Mexican population. The percentage of CVD and IHD deaths attributable to low PUFAs Ω-3 intake was 8.1% and 13.0% respectively. The contribution of low intake of PUFAs Ω-3 and nuts/seeds to CVD mortality was higher in less developed countries (11.3% and 13.0% India; 8.1% and 13.8% Mexico) compared to the developed countries (0.078% and 8.9% Japan; 6.5% and 10.7% the U.S.A.) [10]. Table 2 and Table 4.

Metabolic risk factors were associated with 77.4% of total CVD deaths, 84.1% of the IHD deaths, and 68.6% of the ischemic stroke mortality in Mexico. High LDL-c ranked second among the metabolic risk factors with major influence on mortality caused by CVD and IHD (just after high systolic blood pressure), with a contribution of 30.0% and 44.6% to the deaths from these conditions in Mexican individuals. This lipid abnormality ranked third among metabolic risk factors that contributed most to ischemic stroke mortality, with 20.7% of its deaths. In every single selected country, the contribution of the high LDL-c to total CVD deaths was equal to or higher than 15.9%, and in Mexico it had the highest contribution [10]. Table 2 and Table 4.

Probability of premature death by CVD and other chronic diseases in 2016 was of 23.3%–22.5% for low-middle HDI countries, 17.0–15.7% for high HDI countries, and of 14.6%–8.4% for very high HDI countries [30]. From 1990 to 2017, a relative decrease in age-standardized CVD mortality rates of 11.1%, 27.3% 22.0%, 21.2%, 47.9%, 56.8%, and 41.0% in India, Nigeria, Mexico, China, Brazil, Japan, and the U.S.A., was observed respectively [30] (Table 2).

### 3.5. Attributable Expenditures to CVD

In 2006, CVD care concentrated 55% of the total public and private health expenditures directed to address diabetes, obesity, and CVD in Mexico. The expenditures on CVD in USD-PPP were $812.5 million in the ISSSTE, $1.8 billion in the IMSS, and $343.2 million in the Ministry of Health. The equivalent percentages of the annual budget of these institutions were 23.2%, 11.0%, and 2.4%, respectively. Between 48.4% and 66.0% of the total expenditures on chronic diseases in these three institutions corresponded to CVD. The percentage of the annual budget spent on CVD drugs was 2.8% in the ISSSTE, 1.9% in the IMSS, and 0.2% in the Ministry of Health. The expenditures on control and prevention actions for CVD from the annual budget of the Ministry of Health was 2.3% and 0.02%, respectively [2].

In 2012, the IMSS allocated USD-PPP $1.3 billion and USD-PPP $719.8 million to address hypertension and IHD respectively, including ambulatory consultation, laboratory tests, drugs, and hospitalization expenditures. The Ministry of Health spent USD-PPP $425.9 million on hypertension and USD-PPP $107 million on IHD in the same areas [44]. In 2015, hypertension, myocardial infarction, atrial fibrillation, and heart failure represented an expense of USD-PPP $11.2 billion in Mexico, including the loss of productivity and welfare [45].

The Ministry of Health, IMSS, and ISSSTE were expected to respectively allocate USD-PPP $1.6, $2.7, and $1.1 billion to address the increased demand of health services associated with hypertension in 2016. The expenditures expected for users surpassed the sum of these previous amounts, being USD-PPP $5.6 billion [46]. A similar pattern in the proportion of these expenses among health care providers and users over 60 years old was observed by 2015 [47]. In 2016, the IMSS spent USD-PPP $6.6 million on atorvastatin and USD-PPP $16.1 million on pravastatin, and the average unit price of these statins was USD-PPP $1.1 and USD-PPP $1.08, respectively [29] (Table 5).

**Figure 2:**
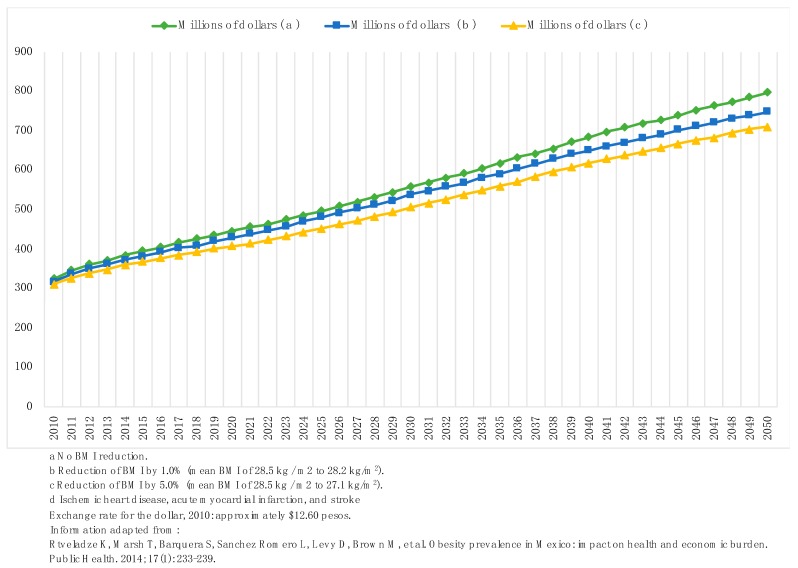
Projected costs of ischemic heart disease, acute myocardial infarction, and stroke. Mexico 2010–2050.

In the case that the average BMI in the Mexican population remains static from 2010 to 2050, the economic burden of IHD, myocardial infarction, and stroke would mean a nationwide accumulated expenditure of USD $23 billion. A reduction of 1.0% in the average BMI in the population would reduce USD $986 million from the accumulated CVD expenditures in this period, while a decrease of 5.0% would save USD $753 million to 20 years and USD $2.1 billion to 40 years [51] (Figure 2).

CVD ranked first among expenditures derived from health care for different causes in India, Mexico, Japan, and the U.S.A., and it represented the second place in China. The highest expenditure due to CVD was reported in the evidence from the U.S.A., USD $316.1 billion in 2013 [55] and $555 billion in 2016 [69]. Projections indicate that the economic losses generated by CVD will be of USD $918 billion [55]–$1.1 trillion [69], USD $756 billion [67], USD $2.17 trillion [66], and of $1.5 trillion [67] for the U.S.A., Japan, India, and China by 2030–2035, respectively. One study in Japan indicated an expected decrease in the economic burden generate by IHD, reducing from USD $15.3 billion in 2017 to USD $11.5 billion by 2029 [71]. Projections were not found for Brazil (Table 2).

### 3.6. Strategies to Decrease the Burden of CVD

According to the WHO, strategies such as reducing high sodium intake, decreasing tobacco use, and optimization of pharmacological treatment, are the Best Buy Interventions against CVD [3]. An additional investment of USD $1.5 per capita per year to scale up these strategies between 2015 and 2030, would mean avoiding the incidence of eight million IHD cases and 13 million strokes in countries where the highest chronic disease burden is concentrated [65].

Studies in some mega-countries have shown how beneficial the implementation of actions similar to the Best Buy Interventions could be to the primary prevention of CVD. For instance, in the U.S.A., adherence to a healthy diet and other lifestyle recommendations are associated with a reduction of up to ≈92% of cardiovascular risk in the population [76,77,78]. In India, a reduction of 3 g/d in average population salt consumption through regulations would prevent ≈400,000 and ≈81,000 CVD events and deaths over 30 years, respectively [63]. Unhealthy food taxation is also a promising strategy against CVD. The projections in Mexico indicate that a reduction of 10% in the consumption of sugar-sweetened beverages through taxes could prevent ≈46,300 CHD cases and 14,200 myocardial infarctions over 10 years [73]. In India, a 10% palm oil tax could reduce ≈363,000 deaths by myocardial infarction in 10 years [64]. Another kind of policy to reduce the CVD burden are the subsidies for healthy food. For example, a 10% reduction in the prices of fruits, vegetables, and nuts/seeds through this strategy, could prevent 0.6% (≈2213), 0.8% (≈2873), and 0.9% (≈3148) of the total annual CHD deaths in the U.S.A., respectively [79] (Table 2).

The control of hypercholesterolemia [80], hypertension [81], and diabetes [80], as well as smoking cessation [82] in the secondary prevention level, are essential to promptly reduce the CVD burden [83]. Studies aimed to evaluate the potential of controlling these risk factors have shown substantial expected benefits. For instance, simulation models in the U.S.A. indicate that optimal achievement of management goals of hypertension could reduce ≈56,000 events and ≈13,000 deaths from CVD annually [75,81]. In China, projections show that optimizing and broadening lipid-lowering therapy coverage could avoid ≈850,000 cases of myocardial infarction and ≈300,000 deaths from CVD each year [54]. In Japan, results derived from a prospective cohort study showed a reduction of ≈66% in the risk of cardiovascular events in healthy adult men associated with smoking cessation for a period equal to or greater than four years [74] (Table 2). However, optimal treatment goals are hard to achieve. In order to effectively treat these conditions, various elements and actions have been recommended: (1) Having a well-trained multidisciplinary health team (e.g., through skills certification); (2) making adjustments to schools’ health curriculums (involving the newest treatment guidelines); (3) incorporating family in therapies; (4) creating mutual-help groups with motivation strategies; (5) improving accessibility and availability of medications; and (6) implementing well-structured interventions (e.g., by using electronic health records) [84].

## 4. Discussion

Compared to developed mega-countries, Mexico and the nations with a similar o lesser HDI experience greater challenges related to CVD. In these countries, dietary risk factors contribute more to cardiovascular mortality. Furthermore, there is a greater probability of premature death from CVD and in the last decades, there has been a lower decrease in mortality rates. The economic burden from CVD was significant in both the developing and developed countries analyzed, representing the main health expenditure. Making this comparison was not possible for Nigeria and Brazil since the information was not identified. Additionally, projections indicate that this economic threat will remain significant for these countries by 2030—especially for China, India, and the U.S.A.

The inverse association between countries’ development and the burden of chronic diseases has also been described at the sub-national level in various countries [85]. In Southern Mexico, there was a greater increase in mortality from diabetes and CVD at the end of the 20th century in comparison to the northern region, which is more developed [6,85]. Because of this, authorities should not only implement a comprehensive strategy, but they should also design targeted strategies that consider the needs of every region. In the southern region, for example, coverage of antihypertensive drugs should be expanded to reduce the high prevalence of adults with hypertension who are not undergoing this treatment [72]. On the other hand, the high prevalence of hypercholesterolemia observed in this study is coupled with low-quality diets that have greater energy density in the northern region [86,87]. This could be mitigated by improving the food environment, dietary recommendations, and increasing availability of and adherence to lipid-lowering treatment in this country area.

This paper gives an overview of the CVD burden in seven mega-countries. Cardiovascular indicators from the GBD study, the WHO Global Health Observatory, national surveys, and from published literature allowed us to compare epidemiological scenarios among nations. Presenting this information, along with CVD economic impact data, provides stakeholders with a comprehensive source of evidence regarding gaps in research, critical epidemiological actions, and financial measures that need to be addressed to counteract CVD. One of the identified barriers in some developing countries is the limited CVD risk monitoring in epidemiological surveillance systems. In Nigeria, for example, there is no representative national data regarding the population’s lipid profile [88]. A similar situation occurs in three of the most important national surveys in India [89]. In Brazil, the population’s lipid concentration has been only evaluated in a few cities [90] and the assessment of hypercholesterolemia in the National Health Survey is limited to self-reporting [43]. Therefore, governments and academic groups from these countries should secure resources to carry out population level studies that describe the prevalence of dyslipidemias. Prioritizing this would allow for a better design, evaluation, and reformulation of strategies to combat CVD.

The development of hypercholesterolemia, hypertriglyceridemia, and hypoalphalipoproteinemia is associated with the presence of obesity and its lifestyle-related determinants [91,92,93,94]. These four CVD risk factors are the most prevalent in Mexico and have been reported to be even greater than in some of the analyzed countries. In Mexico and India, adults with overweight are 5.25 and 4.15 times more likely, respectively, to have mixed dyslipidemias and hypertriglyceridemia [38,95], while up to 95.2% of American individuals with obesity have lipid abnormalities [96]. Considering that reductions starting at 1% of the Mexican population’s average BMI would save up to $986 million USD in the next years [51], intensive strategies to improve dietary patterns, foster active lifestyles, and increase access to pharmacological treatments that normalize bodyweight and the lipid profile could be vital to moderate costs generated by CVD in Mexico and the other nations.

Statins are the most utilized medications in Mexico to treat CVD due to their cost-effectiveness [97]. Their average unit cost for Mexican health public institutions is ≈USD-PPP $1 [29]. However, the potential benefit of this treatment for Mexicans’ cardiovascular health is mainly hampered by low adherence to medication, as in other countries [98,99,100]. In addition, low nutritional quality diets [16,18,101] and the high prevalence of physical inactivity and sedentarism [102,103] in the country are major constraints for the statins’ positive effects. Inaccurate classification of cardiovascular risk profiles by medical professionals and the lack of diagnosis, are other significant challenges to treatment. This leads to poor management of high-risk patients or extraneous therapies in lower-risk subjects that generate unnecessary costs [104]. This practice, in addition to high prescribed doses of statins and having a high cardiovascular risk profile, have been associated with the non-compliance of the LDL-c goals in Mexico, Brazil, and India [104].

These challenges highlight the need to explore new care and treatment options, especially for population groups with the greatest cardiovascular risk. One alternative includes pharmaceuticals that enhance the receptor activity of the LDL-c molecules and its uptake from plasma to the cells. Until now, these drugs have been indicated in a small proportion of cases and have begun to be studied in clinical randomized control trials [105,106]. Nonetheless, due to their approximate cost/person/year of $14,000 USD, these pharmaceuticals are not cost-effective in clinical practice [107,108]. Given the high number of individuals in the population who are struggling with the affordability of CVD medicines, including aspirin, β blockers, angiotensin-converting enzyme inhibitors, and statins in mega-countries, such as Brazil, China, and India [109], cheaper drugs are still a priority. A lower-cost option is a polypill made from the combination of active substances that produce drug synergism. Its use and formulation with patent-free substances have been proposed as strategies to improve adherence and cost-effectiveness of treatment for CVD [110,111,112,113,114,115,116,117,118]. However, consistent evidence is needed to support generalized use.

Due to the lack of comparable studies on CVD-related expenditures, the economic burden produced by these diseases cannot be standardized or easily characterized in the analyzed countries. Data from developed countries are obtained through comprehensive economic analyses, but with distinct methodologies and indicators. In estimates from the U.S.A., for example, costs were considered from hospital care, ambulance and emergency room, nursing services, and medications. In contrast, estimates from Japan were obtained from a study which adapted the Cost of Illness method, taking into account direct costs of medical attention, morbidity, mortality, and the long-term costs and economic burden for families [53,59]. In Mexico, four recent studies developed with different comprehensive methods, national registries, and population-based datasets cover economic estimates for hypertension, IHD, heart failure, myocardial infarction, and atrial fibrillation from 2012 to 2017 [44,45,46,47]. On the other hand, although a considerable number of studies from India and Nigeria at the regional level focused on catastrophic health expenditures or micro-costing approaches was identified, the nationwide current information on total CVD-related costs was limited. Methodological frameworks of some of these studies were not clearly reported (see Supplementary file 2).

Despite this heterogeneity, it is possible to suggest that the financial burden of CVD in developing countries may be higher than reported if all types of costs were considered. For example, estimates in Mexico indicate that four kinds of these diseases consumed approximately USD-PPP $11.2 billion in 2015 or the equivalent to ≈4% of the total health expenses in the country [45]. Although this study, considerably similar to another in Brazil [68], uses direct costs such as patients’ hospital care and the loss of economic productivity, these statistics and those from previous years would increase if out-of-pocket expenditures were included, which represent 44% of the total health expenditure and more than 50% of total expenses associated with hypertension in Mexican adults [46,47]. Out-of-pocket CVD expenses for 15 months in countries such as China and India have been reported to be 15.0%–40.1% and 39.3%–54.9% of annual household expenses, respectively [119]. In addition, catastrophic health expenditure in some of the analyzed mega-countries is considerably higher in households where family members with hypertension live [120]; further, it is significantly high, even for insured individuals [121]. In any case, the financial stress due to CVD experienced by families should be considered in future comprehensive studies.

Management of CVD is a large-scale challenge. In 2006, ≈60% of Mexican adults required interventions to reduce their cardiovascular risk [31]. Indeed, this prevalence could be greater considering current population ageing trends and CVD treatment updated guidelines. Optimal treatment for current CVD cases is key for diminishing the cardiovascular burden in the short term [84]. However, deficiencies in health systems, the complexity of illness, and the high cost of secondary care [84,122] make it difficult to effectively treat CVD in countries such as Mexico [84]. Because of this, authorities from each country should opt to allocate resources to a systematic strategy focused on improving capacity of health care personnel through modifications to university study plans that encourage of lifelong learning, strengthening family inclusion and motivation techniques in medical care, and on guaranteeing technological infrastructure and drugs for adequate management and treatment of patients.

The CVD burden should also be addressed through effective interventions based on the life-course approach and environmental strategies. The modification of the food system through taxes to sugar sweetened beverages (SSBs), an understandable front-of-pack nutrition label, and subsidies for healthy foods are complementary components to prevent cardiovascular risk factors, CVD, and its comorbidities in the next generations. They favor healthy diets and facilitate informed consumption decisions in general and at-risk population [3,123,124]. Furthermore, the SSBs tax has been shown to reduce sales of these products in Mexico and other countries [125,126,127,128,129,130], which in the long-term could decrease cardiovascular risk factors such as high BMI and hyperglycemia, bringing considerable economic benefits. Accordingly, this fiscal measure should be considered a policy with great potential if it is adopted as a CVD prevention strategy.

## 5. Conclusions

The findings of this study have implications for future research and public health actions related to CVD in the analyzed countries. In general, this study highlights for international congruency in methods and implementation practices in order to more effectively compare across countries, specifically to identify the attributable economic impact of CVD in these nations, considering expenditures absorbed by institutions and families. Public health implications are evident considering that CVD currently generate a significant health and economic burden around the world, particularly in Mexico and six other mega-countries that hold approximately 50% of the global population. Although the increase of morbidity and cardiovascular mortality in the developed countries from this group of nations has slowed in the last years, projections show that costs related to CVD will remain significant in the next decades. Consequently, investment in strategies and prevention policies must continue and be raised in order to reduce the incidence of these diseases and their risk factors in developed mega-countries.

Developing mega-countries face greater obstacles due to CVD than developed countries. They also experience shortcomings in clinical practice associated with failure to reach lipid profile targets and deficiencies in epidemiological surveillance instruments that limit cardiovascular monitoring in the population. This suggests an urgent need to implement well-structured interventions at the primary and secondary care level in order to optimize diagnosis, the prescription of pharmacological treatment and lifestyles, and the adherence to these interventions, especially in countries such as Mexico and India, where higher prevalence of some dyslipidemias are observed. The evaluation of dyslipidemias in the Nigerian and Brazilian population through national representative studies must be strengthened. Governments from developing mega-countries must centralize their efforts so that the food system does not favor the obesogenic environment and exacerbate the high contribution that dietary risk factors already have on CVD mortality rates, as this would translate into a substantial reduction in the economic impact and health burden caused by these diseases in the next generations. The loss of sustainable development that CVD would eventually cause to the most populated countries in the world will only be mitigated through the implementation of cost-effective strategies at all the medical care levels and by adopting a preventive health approach.

## Figures and Tables

**Table 1:** Prevalence of cardiovascular diseases (CVD) risk factors according to Mexican National Surveys, 2006 and 2012.

	México		North		Center		Center-West		South	
	2006 ^b^	2012 ^c^	2006 ^b^	2012 ^c^	2006 ^b^	2012 ^c^	2006 ^b^	2012 ^c^	2006 ^b^	2012 ^c^
Metabolic conditions associated to CVD risk, prevalence (%)										
Overweight and obesity defined by BMI ^a^	69.7	71.3	71.5	72.8	70.2	71.2	69.3	70.2	66.6	71.0
Abdominal obesity ^a^	75.7	74.0	79.7	76.6	74.6	76.4	78.1	74.7	71.7	71.6
Hypertension ^a^	31.6	31.5	34.2	36.5	30.1	29.8	33.5	32.3	30.0	28.0
Hypertriglyceridemia ^a^	31.5 [9]	--	29.2	--	42.0	--	28.2	--	22.4	--
Hypercholesterolemia ^a^	43.6 [9]	--	46.3	--	52.1	--	42.1	--	29.8	--
Hypoalphalipoproteinemia ^a^	60.5 [9]	--	58.3	--	49.3	--	65.9	--	72.8	--
High LDL-c ^a^	46.0 [9]	--	--	--	--	--	--	--	--	--

^a^ Overweight and obesity defined as body mass index (BMI) ≥25 kg/m^2^ and abdominal obesity as a waist circumference ≥80 cm for women and ≥90 cm for men; hypertension defined as systolic/diastolic blood pressure ≥140/≥90 mm Hg; hypertriglyceridemia defined as triglycerides ≥150 mg/dL, hypercholesterolemia as total cholesterol ≥200 mg/dL, hypoalphalipoproteinemia as high-density lipoprotein cholesterol <50 mg/dL for women and <40 mg/dL for men and high low-density lipoprotein cholesterol (LDL-c) concentration as low-density lipoprotein cholesterol ≥130 mg/dL. ^b^ National Survey of Health and Nutrition 2006 (Spanish acronym: ENSANUT 2006). ^c^ National Survey of Health and Nutrition 2012 (Spanish acronym: ENSANUT 2012).

**Table 2:** Epidemiological and economic scenario related to CVD in seven mega-countries.

	Low-Medium HDI		High HDI			Very High HDI	
	India	Nigeria	México	China	Brazil	Japan	USA
Hypertriglyceridemia, % ^a^	29.5 [38]	23.4 [39]	31.5 [9]	13.8 [41]–17.7 [40]	23.2 (women) 40.7 (men) [42]	18.0 [36,37]	30.0 [34]
Hypercholesterolemia, % ^a^	13.9 [38]	25.9 [39]	43.6 [9]	6.9 [41]–10.1 [40]	12.5 (self-report) [43]	16.2 [36,37]	12.4 [33]
Hypoalphalipoproteinemia, % ^a^	72.3 [38]	43.8 [39]	60.5 [9]	11.0 [40]–20.4 [41]	20.7 (women) 14.7 (men) [42]	12.7 [36,37]	18.4 [33]
High LDL-c, % ^a^	11.8 [38]	-	46.0 [9]	8.1 [41]–8.8 [40]	57.6 (women) 58.5 (men) [42]	11.1 [36,37]	27.0 [35]
Probability of dying of CVD, % ^b^							
2000	26.6	25.5	16.8	21.5	24.4	11.4	18.0
2016	23.3	22.5	15.7	17.0	16.6	8.4	14.6
Age-standardized CVD deaths per 100,000 inhabitants ^c^							
1990	317.7	248.8	195.8	332.3	341.8	183.7	256.1
2017	282.3	181.0	152.8	261.9	178.0	79.4	151.1
% Change	−11.1	−27.3	−22.0	−21.2	−47.9	−56.8	-41.0
CVD mortality attributable to risk factors in 2017, % ^c^							
Low intake of nuts and seeds	13.0 [8.2, 18.4]	7.3 [4.2, 10.9]	13.8 [8.8, 19.4]	8.7 [5.5, 12.2]	10.8 [6.9, 15.0]	8.9 [5.6, 12.6]	10.7 [6.4, 15.4]
Low intake of polyunsaturated fatty acids (PUFAs) (Ω-3)	11.3 [5.3, 18.3]	7.8 [3.5, 12.6]	8.1 [3.6, 14.0]	6.3 [2.9, 10.5]	5.1 [2.1, 9.0]	0.078 [0.006, 0.27]	6.5 [2.7, 11.7]
High systolic blood pressure	53.0 [47.1, 58.9]	58.6 [52.2, 64.5]	49.7 [42.6, 57.0]	54.5 [47.9, 61.0]	53.3 [47.9, 58.8]	49.4 [43.1, 55.6]	44.5 [37.8, 51.1]
High LDL cholesterol	24.1 [18.4, 30.2]	15.9 [11.0, 22.0]	30.0 [22.5, 38.1]	19.6 [14.2, 26.2]	25.1 [20.0, 30.6]	22.1 [16.2, 29.2]	25.1 [18.8, 32.1]
Expenditure on CVD nationwide ^d^							
*Total*, $USD	Cumulative gross domestic product loss associated with coronary heart disease, stroke, and diabetes (2006–2015) $16.7 billion [61]	Cumulative gross domestic product loss associated with coronary heart disease, stroke, and diabetes (2006–2015) $1.17 billion [61]	Hypertension, myocardial infarction, atrial fibrillation and heart failure only [45]: 2016 USD- purchasing power parities (PPP) 11.3 billion (2015)	Hospitalization expenses related to acute myocardial infarction, intracranial hemorrhage, and cerebral infarction: $13.2 billion (2014) [57]	$11.1 billion (2015) [60] Economic impact of hypertension, heart failure, myocardial infarction, and atrial fibrillation $17.4 billion [68] (2015)	$109.6 billion (2014) [59] Costs associated with ischemic heart disease: $16.13 billion (2014) [71]	$316.1 billion (2013) [55] CVD: $555 billion (2016) [69] Costs of informal caregiving for patients with CVD: $61 billion (2015) [70]
*Rank nationwide ^e^*	First (among projected economic losses due to CVD, diabetes, chronic respiratory disease, cancer, and mental health conditions to 2030) [66]	-	First (among CVD, obesity and diabetes expenditures) [2]	Second (among projected expenditures due to CVD, diabetes, chronic respiratory disease, cancer, and mental health conditions to 2030 [67]	-	First (among expenditures generated by cancer, heart disease, and cerebrovascular disease) [59]	First (among expenditures generated by 14 aggregated condition categories) [53]
*Fraction of different relevant economic indicators spent on CVD nationwide, %*	-	-	Hypertension and ischemic heart disease (IHD) only (2012) [44]: 2% from the Ministry of Health’s total medical expenditure 8.1% from I Mexican Social Security Institute (Spanish acronym: IMSS)’ total medical expenditure 0.13% from GDP Hypertension, myocardial infarction, atrial fibrillation and heart failure (2015) [45]: 4% from total medical expenditure	-	0.7% from GDP (2010–2015) [60]	-	11% from total medical expenditure (2013) [53]
Projected expenditure on CVD, $USD ^d^	Economic losses due CVD: $2.17 trillion (2012–2030) [66]	Cumulative gross domestic product loss associated with coronary heart disease, stroke, and diabetes: $1.17 billion (2006–2015) [61]	$9.3 billion (2010–2030) $23.5 billion (2010–2050) [51]	Economic losses due to CVD: $1.5 trillion (2010–2030) [67]	-	Economic losses due CVD $756 billion (2010–2030) [67] Predicted cost associated with ischemic heart disease [71]: $15.3 billion (2017) $11.5 billion (2029)	$918 billion (2030) [55] CVD: $1.1 trillion (2035) [69] Costs of informal caregiving for patients with CVD: $128 billion (2035) [70]
Specific estimates of potential savings ($USD) or diminishable burden of CVD due to prevention and control policies ^d^	20% taxation on palm oil purchases in 10 y [64]: ≈363,000 deaths from myocardial infarctions (1.3% absolute reduction in CVD deaths) Reduction in salt intake by 3 g/d in 30 y [63]: ≈400,000/81,000 CVD events/deaths	-	Reduction on averaged IMC (2010-50): <1% $986 million<5% $2.1 billion [51] A 10% reduction in consumption of sweetened-sugar beverages in 10 y [73]: 46,300/9300 coronary heart disease cases/deaths 6200/1600 stroke cases/deaths 14,200 myocardial infarctions	Extension and optimization of coverage of lipid-lowering and antihypertensive treatments, (2016–2030) [54]: $932 billions 850,000 cases of myocardial infarction 300,000 CVD deaths Reducing CVD mortality by 1% per year (2010–2040): $10.7 trillion [58]	-	Reduction of ≈66% in the risk of cardiovascular events, associated to quitting smoking for four years or more in healthy adult men [74]	Investment of $10 USD/person/year in community programs to address lifestyle modifiable risk factors [56]: $18 billion USD Optimal compliance with guidelines for the management of hypertension: Annual reduction of 56,000 events and 13,000 CVD deaths [75]

^a^ Data of dyslipidemias prevalence was obtained from population-based studies or reviews from each country. ^b^ Probability (%) of dying between age 30 and exact age 70 from any of cardiovascular disease, cancer, diabetes, or chronic respiratory disease (Global strategy for women’s, children’s and adolescents’ health), indicator from Global Health Observatory, World Health Organization. ^c^ Data from the Institute for Health Metrics and Evaluation (IHME). Global Burden of Disease (GBD) Compare. Seattle, WA: IHME, University of Washington, 2017. ^d^ Estimates among countries can variate according to methodologies and designs from the information sources. ^e^ Rank among different causes included in the information sources.

**Table 3:** Prevalence of Mexican adult candidates for treatments according to cardiovascular risk profiles, 2006.

Cardiovascular Risk Profile		LDL (mg/dL), Ranges (%)						Lifestyle Changes Treatment	Pharmacological Treatment
		<70	70–99.9	100–129.9	130–159.9	160–189.9	>190	Prevalence (%)	Prevalence (%)
	Total Prevalence (%)	7.3	18.4	28.6	21.4	13.1	11.2	36.3	24.2
Profile 1: coronary heart disease or equivalent condition (acute myocardial infarction, stroke, non-traumatic amputation or diabetes) [31]									
Coronary heart disease or equivalent condition	13.8	10.1	19.4	21.4	22.5	15.3	11.3	70.5	
Diabetes	12.5							71.4	
Coronary heart disease, stroke, non-traumatic amputation	1.3							74.4	
Without coronary heart disease or equivalent condition [31]									
Two or more CVD risk factors (age ≥ 45 years for men and ≥55 years for women, family history of coronary disease, current smoking or hypertension)	31.5	8.2	23.1	30.8	19.4	10.6	7.9	38.6	23.9
Profile 2: ≥2 CVD risk factors + Framingham score <10%	23.3	9.6	24.9	34.7	19.8	7.0	3.9	30.7	10.9
Profile 3: ≥2 CVD risk factors + Framingham score 10–20%	5.9	4.0	18.4	22.2	21.3	18.8	15.2	55.3	55.3
Profile 4: ≥2 CVD risk factors + Framingham score >20%	2.2	3.8	15.8	12.9	10.1	27.2	30.3	80.5	80.5

Information adapted from: Gómez-Pérez F, Rojas R, Villalpando S, Barquera S, Rull J, Aguilar-Salinas C. Prevention of cardiovascular disease based on lipid lowering treatment: a challenge for the Mexican health system. Salud Publica De Mexico. 2010; 52 Suppl 1S54-S62 [31].

**Table 4:** Age-standardized deaths of CVDs attributable to risk factors in Mexico, 2017.

CVD Risk Factors	Cardiovascular Diseases ^a^, Deaths per 100,000 Inhabitants [Uncertainty Interval] (%) ^b^	Ischemic Heart Disease, Deaths per 100,000 Inhabitants [Uncertainty Interval] (%) ^b^	Ischemic Stroke, Deaths per 100,000 Inhabitants [Uncertainty Interval] (%) ^b^
Attributable Burden of Dietary Risk Factors			
Total	73.9 [65.3, 83.5] (48.4)	63.0 [54.6, 71.5] (65.7)	3.4 [2.5, 4.4] (23.2)
Low intake of nuts and seeds	21.1 [13.4, 29.7] (13.8)	21.1 [13.4, 29.7] (22.0)	.
Low intake of vegetables	17.5 [8.6, 28.3] (11.5)	14.4 [5.7, 24.6] (15.1)	1.0 [0.3, 1.8] (6.7)
Low intake of PUFAs (Ω-3) from fish and seafood	12.4 [5.5, 21.2] (8.1)	12.4 [5.5, 21.2] (13.0)	.
Low intake of fruits	11.3 [5.3, 19.1] (7.4)	7.0 [2.2, 13.5] (7.3)	1.4 [0.6, 2.3] (9.3)
High intake of trans fatty acids	10.2 [6.0, 15.9] (6.7)	10.2 [6.0, 15.9] (10.7)	.
High intake of sodium	7.2 [0.1, 22.4] (4.7)	4.6 [0.1, 14.3] (4.8)	0.6 [0.01, 1.9] (3.9)
Low intake of fiber	4.2 [1.8, 7.6] (2.7)	4.2 [1.8, 7.6] (4.3)	.
Low intake of polyunsaturated fatty acids	7.8 [3.3, 13.1] (5.1)	7.8 [3.3, 13.1] (8.2)	.
High intake of processed meat	2.4 [0.1, 4.9] (1.6)	2.4 [0.1, 4.9] (2.5)	.
Low intake of legumes	1.8 [0.5, 4.1] (1.2)	1.8 [0.5, 4.1] (1.9)	.
High intake of sugar-sweetened beverages	8.6 [-] (5.6)	8.6 [-] (8.9)	.
Low intake of whole grains	11.5 [5.7, 18.9] (7.5)	8.8 [4.2, 15.1] (9.1)	0.9 [0.5, 1.5] (6.2)
Attributable Burden of Metabolic Risk Factors			
Total	118.3 [109.4, 126.6] (77.4)	80.6 [72.9, 88] (84.1)	10.1 [8.2, 12.2] (68.6)
High systolic blood pressure	75.9 [65.0, 87.0] (49.7)	46.6 [36.4, 56.8] (48.6)	6.2 [4.5, 8.0] (41.9)
High LDL cholesterol	45.8 [34.5, 58.2] (30.0)	42.7 [31.6, 54.4] (44.6)	3.0 [1.0, 6.4] (20.7)
High fasting plasma glucose	46.6 [30.6, 68.2] (30.5)	35.4 [20.4, 55.9] (37.0)	4.6 [2.2, 9.9] (31.2)
Overweight and obesity	36.5 [23.1, 50.3] (23.9)	22.5 [13.5, 32.2] (23.5)	2.1 [1.2, 3.2] (14.3)
Impaired kidney function	17.2 [14.5, 20.2] (11.3)	13.7 [11.2, 16.4] (14.3)	1.6 [1.1, 2.2] (10.9)

^a^ It includes all CVDs considered by Global Burden of Disease Study by 2017. ^b^ It refers to the attributable percentage from risk factors to the total of CVD deaths. Information adapted from: Institute for Health Metrics and Evaluation (IHME). GBD Compare Data Visualization. Seattle, WA: IHME, University of Washington, 2017. Available from http://vizhub.healthdata.org/gbd-compare. Accessed 20 November 2018.

**Table 5:** Estimated expenditures for CVD in Mexico expressed in USD-PPP and pesos ^a^, 2006–2016.

Author	Year	CVD/Item	Institutions	Total Expenditure Nationwide	
				USD-PPP	Pesos
Ávila-Burgos et al. [2]	2006	Ischemic heart disease, cerebrovascular diseases, hypertension, peripheral vascular diseases, rheumatic diseases and rheumatic heart diseases, congestive heart failure, pulmonary heart disease and other heart disease	Ministry of Health	343,152,856 (48.4% ^b^) (2.4% ^c^)	3,043,422,684
			IMSS	1,766,349,589 (61.3 ^b^) (11.0% ^c^)	15,665,754,501
			Institute for Social Security and Services for State Workers (Spanish acronym: ISSSTE)	812,492,790 (66.0% ^b^) (23.2 ^c^)	7,205,998,550
			Private sector	723,092,634 (40.5% ^b^)	6,413,108,571
Figueroa-Lara et al. [44]	2012	Hypertension	Ministry of Health	425,901,122	3,777,317,057
			IMSS	1,312,998,401	11,644,982,822
		Ischemic heart disease	Ministry of Health	107,039,658	949,334,732
			IMSS	719,802,391	6,383,927,403
		Both	Ministry of Health	532,940,781(2% ^c^)	4,726,651,789
			IMSS	2,032,800,792(8.1% ^c^)	18,028,910,225
Stevens B et al. [45]	2015	Hypertension	Complete health system including loss of productivity and welfare	2,645,591,792	23463753600
		Heart failure		3,148,115,549	27,920,636,800
		Myocardial infarction		4,550,683,595	40,360,012,800
		Atrial fibrillation		973,931,131	8,637,795,200
		Total		11,233,946,555 (4% ^d^)	99,633,872,000
Arredondo et al. [47]	2015	Hypertension (adults over 60 years old)	Ministry of Health	585,138,637.9	5,189,594,579
			IMSS	985,625,322.1	8,741,510,981
			ISSSTE	394,118,027	3,495,432,782
			Users	2,051,383,788	18,193,722,816
			Total	4,022,328,467	35,674,031,175
Arredondo et al. [46]	2016	Hypertension	Ministry of Health	1,604,264,783	14,228,224,358
			IMSS	2,674,376,729	23,719,047,208
			ISSSTE	1,069,487,777	9,485,287,094
			Users	5,566,411,341	49,368,502,187
			Total	10,914,540,614	96,801,060,709
IMSS datasets [29]	2016	Total expenditure on statins by IMSS in 2016	Estimates from IMSS datasets		
		Atorvastatin		6,594,810	58,489,369
		Pravastatin		16,076,150	142,579,376
		Average unit price in 2016			
		Atorvastatin		1.1	9.9
		Pravastatin		1.08	9.6

^a^ Estimate expenditures were deflated considering cumulative inflation until the year of the most recent study: 2006−2016 = 46.81%; 2008−2016 = 32.82%; 2014−2016 = 5.56% and 2015−2016 = 3.36%. Then, estimates were adjusted by purchasing power parities (PPP) for 2016 (1 USD-PPP = 8.87 pesos). ^b^ Fraction from total chronic disease expenditures spent on CVD, %. ^c^ Fraction from total budget per institution spent on CVD, %. ^d^ Fraction from total healthcare expenditure in Mexico.

## Supplementary Materials

The following are available online at https://www.mdpi.com/1660-4601/16/20/4041/s1, PRISMA checklist (see Supplementary file 1), Supplementary Table S1 (see Supplementary file 2), and Supplementary Tables S2–S5 (see Supplementary file 2).
